# Mixed-strain housing for female C57BL/6, DBA/2, and BALB/c mice: validating a split-plot design that promotes refinement and reduction

**DOI:** 10.1186/s12874-016-0113-7

**Published:** 2016-01-27

**Authors:** Michael Walker, Carole Fureix, Rupert Palme, Jonathan A. Newman, Jamie Ahloy Dallaire, Georgia Mason

**Affiliations:** 1 Animal Biosciences, University of Guelph, Guelph, ON N1G 2 W1 Canada; 2 Department of Biomedical Sciences/Biochemistry, University of Veterinary Medicine, Veterinärplatz 1, Vienna, A-1210 Austria; 3 College of Biological Science, University of Guelph, Guelph, ON N1G 2 W1 Canada

**Keywords:** Mice, Refinement, Reduction, Animal Welfare, Experimental Design, Split-Plot

## Abstract

**Background:**

Inefficient experimental designs are common in animal-based biomedical research, wasting resources and potentially leading to unreplicable results. Here we illustrate the intrinsic statistical power of split-plot designs, wherein three or more sub-units (e.g. individual subjects) differing in a variable of interest (e.g. genotype) share an experimental unit (e.g. a cage or litter) to which a treatment is applied (e.g. a drug, diet, or cage manipulation). We also empirically validate one example of such a design, mixing different mouse strains -- C57BL/6, DBA/2, and BALB/c -- within cages varying in degree of enrichment. As well as boosting statistical power, no other manipulations are needed for individual identification if co-housed strains are differentially pigmented, so also sparing mice from stressful marking procedures.

**Methods:**

The validation involved housing 240 females from weaning to 5 months of age in single- or mixed- strain trios, in cages allocated to enriched or standard treatments. Mice were screened for a range of 26 commonly-measured behavioural, physiological and haematological variables.

**Results:**

Living in mixed-strain trios did not compromise mouse welfare (assessed via corticosterone metabolite output, stereotypic behaviour, signs of aggression, and other variables). It also did not alter the direction or magnitude of any strain- or enrichment-typical difference across the 26 measured variables, or increase variance in the data: indeed variance was significantly decreased by mixed- strain housing. Furthermore, using Monte Carlo simulations to quantify the statistical power benefits of this approach over a conventional design demonstrated that for our effect sizes, the split- plot design would require significantly fewer mice (under half in most cases) to achieve a power of 80 %.

**Conclusions:**

Mixed-strain housing allows several strains to be tested at once, and potentially refines traditional marking practices for research mice. Furthermore, it dramatically illustrates the enhanced statistical power of split-plot designs, allowing many fewer animals to be used. More powerful designs can also increase the chances of replicable findings, and increase the ability of small-scale studies to yield significant results. Using mixed-strain housing for female C57BL/6, DBA/2 and BALB/c mice is therefore an effective, efficient way to promote both refinement and the reduction of animal-use in research.

**Electronic supplementary material:**

The online version of this article (doi:10.1186/s12874-016-0113-7) contains supplementary material, which is available to authorized users.

## Background

The 3Rs of refinement, reduction and replacement [1] are widely recommended guidelines for laboratory animal research, which biologists worldwide have to comply with for ethical review purposes (e.g. [2, 3]). Refinements, methods that minimize animal distress, are developed and applied to reduce welfare costs to individual animals (e.g. [4]). Reductions in animal numbers can be achieved by using replacement technologies (e.g. [5]), or, instead, by using more efficient experimental designs (e.g. [6]). Despite this fact, a recent survey found that over 33 % of animal-based studies use inefficient experimental designs [6]. Split-plot designs exemplify how statistical power and efficiency could be increased, so potentially permitting the use of fewer subjects [7, 8]. Here, individual subjects (‘sub-units’) differing in a variable of interest (e.g. in genotype, health status, or individual-level treatment) share an experimental unit (e.g. a cage, mother or litter) to which a treatment is applied (e.g. a drug, diet, or environmental enrichment). How such designs increase the inherent statistical power gained from a given number of animals is detailed below. Such designs are as yet in little used biomedical research, despite their benefits.

We chose to empirically investigate the potential value of split-plot designs by housing mice of varied genotypes (strains) together within enriched or non-enriched cages. Using multiple mouse strains is useful for increasing a study’s external validity: how well results generalize to other environmental contexts, populations or species [9]. High external validity is essential for efficient, useful research, especially given the translational nature of many rodent studies. Working with multiple strains is one way to achieve this [10], because strains show well-documented differences in numerous behavioural and physiological phenotypes (e.g. [11–13]): variation ideal for testing the robustness and generalizability of phenomena under study [14]. Using different strains may also reveal valuable insights into how any treatment effects interact with genotype (e.g. [15]). Conventionally, researchers using multiple strains to reap these benefits would house them all in single-strain cages, like genotypes with like. The different strains would then be either tested sequentially in separate studies, or better, studied in parallel in a factorial design.

Mixed-strain housing, in contrast, in which individuals from different strains are instead caged together, yields all the advantages of testing multiple strains, but in a more statistically efficient way (as long as three or more strains are used). In a mixed-strain housing design, the physical cage is a ‘plot’ that is ‘split’ by including mice from different genotypes (sub-plots) within it (the same way one field [plot] could be ‘split’ by planting different crops within it [sub-plots], e.g. [16]). Why this spilt-plot design is more statistically powerful can be summarized as follows. One factor affecting an experiment’s power – its ability to detect effects – is the number of replicates per treatment group. Due to the lack of independence between mice in a cage, the cage is the independent unit of replication; and if several strains are to be studied, when only one strain is housed per cage (the conventional design), the total number of cages (replicates) must be divided between these strains. In contrast, if these strains are mixed within each cage, then each cage provides replication for every strain. This is what yields the split-plot design’s greater statistical power.

**Fig. 1:**
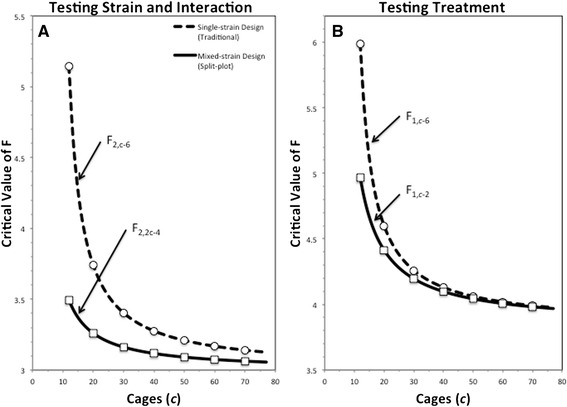
Shown are the critical values of *F* needed to reject the null hypothesis at the *α* = 0.05 level for two hypothetical experiments testing the effects of a treatment vs. a control on three strains of mice. Smaller values for the critical *F* imply greater statistical power. The graphs start at 12 cages, as this is the fewest number of cages that can be used in a balanced, single-strain design with at least two replicates. A) illustrates strain and strain*treatment effects and B) illustrates treatment effects (e.g. enrichment)

A simple way to more formally compare the inherent relative power of these two types of design, all else being equal, is to compare the size of the critical F- value needed to reject the null hypothesis, as shown in Fig. 1 (with lower critical values obviously representing greater power, because they mean that smaller effects can yield statistical significance). As can be seen in the figure, the mixed-strain design has greater power (lower critical values for F), especially when relatively few cages are used, and particularly for strain and strain*treatment interaction effects. More specifically, the differences between the two competing designs arise from the degrees of freedom associated with the mean square used to form each *F*-ratio’s denominator. The single-strain case is a full factorial design [17], where the denominator mean square is the same for all *F*-ratios: the mean square for cages nested in treatment and strain. Thus when two treatments and three strains are used in a conventional design (as in our case), the denominator degrees of freedom is the total number of cages (*c*) minus six (see Additional file 1 for details). The mixed-strain, split-plot design, in contrast, uses two denominator mean squares: one for testing the treatment effect (sometimes called the ‘whole- plot error’), one for testing the effects of strain and the strain by treatment interaction (sometimes called the ‘sub-plot error’) [17]. For testing treatment effects, the mean square for the cages nested in treatment term is used as the denominator, and its degrees of freedom would be the number of cages minus two. For testing the effect of strain or the strain by treatment interaction, the appropriate denominator is the sub-plot error with degrees of freedom equal to two times the number of cages minus four (see Additional file 1 for details). Therefore, all else being equal, the advantage of the split-plot design is that the power to detect effects of the sub-plot (in this case strain) and its interaction with the whole- plot (e.g. enrichment) is greatly increased (particularly at smaller sample sizes). The power to detect whole plot effects is also slightly increased (see Fig. 1; see also Additional file 1: Figures S3 & S4).

Because this type of split-plot design (illustrated here by co-housing strains) is more statistically powerful, it can benefit researchers in two ways. First, for any given sample size and effect size, this design makes it more likely that researchers’ results will be statistically significant (because smaller F-ratios are needed and so smaller effects can be detected: see Additional 1: Figure S3). This can therefore be an excellent way to make the most of small-scale pilot studies, for example. Second, researchers can instead choose to use fewer animals, while retaining the same chance of achieving significance for any given effect size (see Additional file 1: Figure S4). This then allows the principle of reduction to be met (as may be especially advisable when treatments or genotypes have adverse effects on animal welfare): our main interest here.

Furthermore, as well as these statistical benefits, if mixed-strain housing involves mice differing in visual appearance (e.g. coat colour; see Additional file 1: Figure S1), this can confer additional advantages. Individual identification is important for providing a link between each subject and the data they generate. However, all common techniques used for this (e.g. ear notching) negatively impact animal welfare, even if only by causing brief pain [18, 19], and some methods can even lead to confounds in some behavioural research (e.g. [20]). Housing differentially pigmented mice together within cages avoids these problems by allowing them to be easily identified without further manipulation [18]. This also facilitates ease of identification in the home cage [18], potentially making cage-side checks faster and more reliable.

Mixed-strain housing thus potentially has many advantages over traditional, single-strain housing. Previously, we performed a successful ‘proof-of-principle’ using two inbred strains of mice housed together in conventional cages: C57BL/6 (black) and DBA/2 (brown) [18]. Here, we expand upon this by including a third strain, BALB/c (white), as well as an enriched housing condition as a treatment: a common manipulation used in neuroscience and welfare studies. We investigated whether mixed-strain housing modifies the strain-typical phenotypes of the mice, and/or interacts with the effects of enrichment, since this would be problematic. We also assessed whether mixed-strain housing increases the variance in data obtained from the subjects, since this would potentially compromise any statistical power gained through the use of the split-plot design. In addition, we evaluated mouse well-being, to check that co-housing strains created no new welfare concerns. To do this, we housed the three strains in either mixed- or single-strain trios, evenly split across standard or enriched cages (see Additional file 1: Figure S2). 240 female mice were housed in same-strain (60 cages; 20 of each strain) or mixed-strain (20 cages) trios, half of which were enriched, from 3–5 weeks of age until approximately 5 months of age. A total of 26 behavioural, physiological, morphological, and haematological variables were measured, chosen to comprehensively assess strain-typical phenotypes, enrichment effects and also animal welfare.

## Methods

### Animals and housing

240 unrelated female mice were purchased from Charles River Labs at three to five weeks of age. Building upon our previous work co-housing C57BL/6 and DBA/2 females [18], we used 80 mice from each of three strains, C57BL/6, DBA/2, and BALB/c. We chose these for their coat colours and because they are all widely used, comparable in body weight [21], and similarly sociable [22]. We used females because they are commonly group housed [23], necessitating individual identification, and because females make up a large proportion (approximately 70 %) of the inbred mice sold by Charles River Labs [18].

Upon arrival, mice were randomly divided into either same-strain (*n* = 60 total, 20 of each strain) or mixed-strain (*n* = 20) trios. Mixed-strain trios consisted of one mouse of each strain. Mice were also split between two housing treatments: standard and enriched. Standard housing (SH) consisted of ‘shoebox’ cages (12H × 27L × 16Wcm; Allentown Inc.) furnished with: corncob bedding, Shepherd Enviro-dri© nesting material, a standard size paper coffee cup, and *ad lib*. food and water (Harlan® Teklad Global Diet [14 % protein]). Enriched housing (EH) consisted of typical rat cages (21Hcmx47Lcmx25Wcm) furnished with all the same items as standard housing plus two running wheels (one stainless steel mesh 5” upright wheel, Ware Manufacturing Inc.; one plastic mouse igloo & ‘fast-trac’ wheel combo; Bio Serv®), a black polyvinyl chloride tunnel (10 cm long, 4 cm diameter), a small paper cup, a Nestlet, tissues, a cloth hammock (a roughly 12x12cm piece of a sock attached to the cage lid via cable ties), and a steel mesh elevated platform (5H × 40L × 4Wcm long) to access the water. The cages were arranged on shelves in a randomized complete block design (i.e. each block had 8 cages in a random order: 1 cage of each strain and 1 mixed - strain cage, times two for standard and enriched housing), and were completely cleaned once a week. The room was kept at 21 °C and 48 % relative humidity and was on a 12-h reverse light schedule (lights out at 10 am). Three days after arrival every mouse was given an injection of Carprofen (5 mg/kg) and 30 min later [24], two mice per trio were ear notched (one left ear, one right ear), while the third mouse underwent a sham notching procedure. Mixed-strain mice were also notched to ensure ear notching was not confounded with Cage Type treatment. Only two mice were notched per group for welfare reasons and in previous work we found no effect of ear notching [18]. For all procedures listed below, mice were handled using either a tunnel if EH or paper cup if SH, from their home cage, in order to minimize the effect of handling [4]. Due to a few instances of malocclusion, severe barbering, and accidental death, our final sample size was 216 mice arranged in the following groups: 8 SH and 9 EH C57BL/6 trios; 9 SH and 9 EH DBA/2 trios; 10 SH and 10 EH BALB/c trios; 9 SH and 8 EH mixed-strain trios.

### Home cage time budgets during the active (dark) phase

Home cage observations began when the mice were approximately 3 months old and were conducted in two four-hour blocks per day (12 pm-4 pm; 5 pm-9 pm) during the dark period for 12 days over a two-week period (no observations were done on cage cleaning days). The silent observer recorded behaviours once per hour using a mixture of focal (used only for determining whether a behaviour was stereotypic or not) and scan sampling [25], and following a previously determined, well-validated ethogram [26]. For analysis, behaviours were pooled into three categories: normal activity (e.g. locomotion, grooming, eating/drinking), inactivity (e.g. standing still, sleeping), and stereotypic behaviour (e.g. route tracing, patterned climbing). Wheel running behaviour was included in the normal activity category. All these behavioural variables were selected to allow comparison with published strain-typical values [11] and for their use in assessing mouse welfare [27].

### Novel object test

Long latencies to make contact with a novel object are typically interpreted as reflecting higher levels of anxiety or neophobia [28]. To assess this, we modified a previously determined protocol [18, 29]. Mice were placed individually into a novel arena and allowed to habituate for one minute. Next, a novel object (a plastic golf ball) was placed into the centre of the arena and the latency of each mouse to make contact with their nose or paw was recorded. This procedure was performed in triplicate; all mice from one cage were tested at the same time, but in a different arena, and watched by a different observer. Arenas were counterbalanced by observer so that each observer was not always scoring mice in the same arena nor scoring the same strain repeatedly in the mixed-strain cages. Objects and arenas were cleaned between every trial to remove any odour cues. The maximum allowed duration was 5 min; any mouse making no contact was given the maximum score (300 s). The test was run over two days, and there was no effect of day on the outcome (F_1,70_ = 0.10, *p* = 0.75).

### Startle response test

Large responses to sudden auditory tones reflect more anxious phenotypes [30]. Acoustic startle responses were assessed using three Kinder Scientific startle boxes (with Startle Monitor software for analysis) [18]. One cage of mice was tested at a time, with each mouse placed individually into a separate startle apparatus so that they could move around but not rear up. The mice were allowed to habituate for six minutes (50 dB white background noise), and then were played a sharp auditory tone (115 dB for 40 ms). The force generated by each mouse immediately prior to the tone was recorded (to account for body weight), as was the force generated by the mouse over the duration of the tone. The startle response was calculated as the maximum force minus the initial force. This test was conducted over three days, again with no effect of day on the outcome (F_2,69_ = 0.52, *p* = 0.60).

### Forced swim test

This test exploits the fact that rodents in an inescapable situation eventually adopt a characteristic immobile posture, which is amplified by stressors, alleviated by antidepressants, and so interpreted as depression-like behaviour [31, 32]. All procedures were similar to those commonly reported in the literature [33, 34]. The testing room and water temperature ranges were chosen in order to limit the risk of mice developing hypothermia under testing. Tests were conducted from 10:30 am to 7:00 pm over four consecutive days. Cages were brought one at a time to a testing room adjacent to the colony room (white light on, ambient temperature maintained at 29 °C). Mice were allowed to habituate to the testing room in their home cage for 5 min and then were placed individually in three side-by-side transparent glass cylinders (height 23 cm, diameter 19 cm), visually separated by opaque screens and filled with 18 cm of water (water temperature: 25.38 °C ± 0.29). Mice were individually videotaped for 6 min (2 min of habituation and 4 min of testing [32]). They were then placed back in their home cage, allowed to recover and dry fully for 20 min and brought to the colony room. Cylinders were cleaned, rinsed with water, and filled again with clean water between each test. Treatments were counter-balanced between days of testing, hour of testing and the three cylinder locations. Two observers (one experimenter [CF] and one assistant blind to the treatments and hypothesis) scored each mouse’s latency to immobility and total duration of immobility out of the 4 min of test. A mouse was judged to be immobile when it remained floating for at least 2 s with at least 3 legs totally motionless [32]. Inter-observer reliability was assessed using a correlation, and was good for both measures (Latency: F_1,208_ = 1110.2, *p* < 0.0001; Duration: F_1,154_ = 1145.0, *p* < 0.0001); data were therefore averaged between observers for each animal for further analysis.

### Baseline faecal corticosterone metabolite assessment

Again, we followed an established protocol for faecal collection and analysis [18]. Faeces were collected from each mouse during the startle response test and during the novel object test. The two samples were pooled and then frozen at −20 °C until processed. The assay has been validated for mice [35], and all details regarding the procedure have been published [36]. Eleven mice did not produce enough faeces for a complete assay, so were excluded from the analysis.

### Body weight

Mice were weighed immediately upon arrival and again just prior to death. We used these values to calculate the growth of the mice over the duration of the experiment (final weight minus initial weight), correcting for initial body weight. Body weight at death was also used as a covariate in the model for spleen weight [18].

### Post mortem measures

Mice were killed via cervical dislocation at approximately 5 months of age by a trained technician. Similar to previous work [18], a blood sample was taken via cardiac puncture immediately after death. A small portion of this sample was used to determine blood glucose, using a Contour® blood glucose meter; the rest of the sample was stored in a heparinized tube. After this, the mouse was dissected and the spleen was removed and weighed. Spleen mass is likely to reflect immune status in mammals (larger spleens suggest better immune function) [37], and also likely differs between our three strains of mice [38]. Blood samples were sent to the University of Guelph Animal Health Laboratory for a Complete Blood Count Analysis. Unfortunately, up to 39 samples (depending on the variable) were lost due to the presence of clots in the sample and therefore could not be analysed.

### Statistical analyses

All analyses were conducted in JMP® 10. Mixed models were used to test all hypotheses and to run the behavioural consistency checks mentioned in the Methods. The model used for each dependent variable was similar:

$$ y= Cage\left( Strain,\; Cage\; Type,\; Enrichment\right)+ Strain+ Cage\; Type+ Enrichment+ Strain* Cage\; Type+ Strain* Enrichment+ Cage\; Type* Enrichment+ Strain* Cage\; Type* Enrichment $$

Strain has three levels, Enrichment has two levels (EH or SH), and Cage Type also has two levels (single- or mixed-strain). Cage was included as a factor in the model in order to avoid pseudoreplication because mice housed in the same cage are non-independent [39, 40], and was set as a random effect (the only one in the model) so that inferences can be made that go beyond just the cages used in this experiment [41]. Strain, Cage Type (single- or mixed-strain), and Enrichment (EH or SH) are nested within Cage. In a few cases, extra terms considered necessary as controls were added to the model (e.g. body weight in the spleen weight analysis) [18]. Data were transformed using Box-Cox transformations where necessary to meet the assumptions of mixed models. If mixed-strain housing alters the phenotypes of the mice, Cage Type would have significant effects; and if mixed-strain housing altered the magnitude of strain differences (arguably a more important concern), Cage Type*Strain would be significant. Similarly, if mixed-strain housing altered the effects of enrichment, Cage Type*Enrichment would be significant. For these specific analyses, 26 models (one for each dependent variable; see Additional file 1: Table S1) were run, generating 182 p-values, all to test the hypothesis that mixed-strain housing affects mouse phenotype. Because multiple comparisons increase the risk of Type 1 errors, and because these were all from the same ‘family’ of analyses (each individual p-value representing a single test of one statistical hypothesis [42]), we performed a correction for the false discovery rate: the step-down multiple hypothesis testing procedure [43]. As a result of this procedure, the threshold for significance for this group of analyses was reduced from 0.05 to 0.0003. Tukey’s tests were then used to investigate any significant differences within categorical variables.

To analyse the impact of mixed-strain housing on the variability of the measures, we ran two additional tests on the standard deviations (SD) of the dependent variables [16]. Firstly, we ran a simple sign test to make pairwise comparisons for every dependent variable between single- and mixed-strain housed mice of each strain and housing treatment (156 comparisons). Secondly, we used the following mixed model to see if mixed-strain housing predicted a difference in standard deviations and whether any differences were due to all or just some of the dependent variables:

$$ SD= Cage\; Type+ Variable+ Strain+ Enrichement+ Cage\; Type* Variable+ Cage\; Type* Strain+ Cage\; Type* Enrichement+ Enrichement* Variable+ Enrichment* Strain+ Cage\; Type* Enrichment* Strain $$

Here, a significant Cage Type effect would indicate that mixed-strain housing impacted the standard deviations of the dependent variables, and a significant Cage Type * Variable interaction would indicate that the standard deviations of some of the dependent variables are affected differently than others by mixed-strain housing. The other factors in the model are included as blocking factors.

### Power and relative efficiency simulations

Because our effects were tested for using a mixed model, statistical power had to be estimated using a simulation approach [44, 45]. We used a custom-written program for the statistical software R [46] with the “nlme” package [47]. The code is archived in the University of Guelph Research Data Repository: http://hdl.handle.net/10864/10939. For these analyses, single- and mixed-strain designs are being considered separately, and then compared to each other. We used the following procedure to estimate power for each combination of dependent variable and experiment. First, we analyzed data using a similar model as used in JMP above:

$$ y= Cage\left( Strain, Enrichment\right)+ Strain+ Enrichment+ Strain* Enrichment $$

We extracted the following sample parameters: coefficients for each strain and housing type; coefficients for each strain-by-housing combination; standard deviation among cages; and standard deviation of residuals. These respectively represent main effects, interaction effects, between-cage error, and between-individual error. Second, we used these parameters to run Monte Carlo simulations: for each combination of dependent variable and experimental design, we randomly generated a series of simulated study samples drawn from a hypothetical population with average characteristics (coefficients and standard deviations) identical to those we observed empirically. In these simulated samples, each animal's value for a dependent variable is the sum of its particular Strain, Enrichment, and Strain*Enrichment coefficients, and random coefficients for cage and individual (the latter two randomly generated from a normal distribution, centred at zero, with the appropriate empirically-observed standard deviation). Third, using the same statistical model as above to analyze each of 100,000 simulated study samples for each dependent variable in each experiment, we calculated power as the fraction of these that produced p-values less than or equal to alpha = 0.05.

To compare *post hoc* power, we estimated power for the sample sizes used in our actual experiments. For those dependent variables that were not obtained from all subjects, we reduced the size of the simulated study populations accordingly. For each type of effect (Strain, Enrichment, Strain*Enrichment), we compared statistical power, estimated by Monte Carlo simulations, across all dependent variables using t-tests. For data from these simulations, we also made sure of the reliability of our estimates by testing for a correlation between power calculated from simulation rounds 1 through 50,000 and power calculated from rounds 50,001 through 100,000. These simulations were consistent (F_1,124_ = 1995227, p < 0.0001; r^2^ = 0.999), and not affected by type of effect – Strain, Enrichment, and Strain*Enrichment -- (F_5,124_ = 0.67, p = 0.64) or by Variable (F_25,124_ = 0.98, *p* = 0.49).

To test relative efficiency (the number of cages necessary to obtain equal power between the two designs), we again used Monte Carlo simulations, this time to compare the sample sizes required to obtain 80 % power between single- and mixed-strain designs. We only tested sample sizes that were runnable as a balanced model in the single- strain design (i.e. multiples of 6, with a minimum of 12). Even though the mixed-strain model could be balanced in multiples of 2, this would not lead to an even comparison between the two designs. For each combination of Cage Type (single or mixed), effect type (Strain, Enrichment, Strain*Enrichment), and dependent variable, we ran 100,000 simulations to estimate power at a variety of sample sizes, until we identified the lowest sample size yielding at least 80 % power. Because this is a very computation-heavy, time-consuming process, we did not estimate the actual N required in cases where it was above a ceiling of 600 cages. We then calculated the median (and inter-quartile range) number of cages needed to achieve 80 % power across all 26 dependent variables. Mann–Whitney U tests (non-parametric) were used to compare single- and mixed- strain designs (Table 1). Any variable that required greater than 600 cages to achieve 80 % power was given the maximum value of 600 for these analyses. Finally, we calculated partial eta squared (η_p_^2^) values as a measure of effect size for every dependent variable, split by effect (Enrichment, Strain, Strain*Enrichment) and by single- or mixed-strain (Additional file 1: Tables S2–S4) [48].

**Table 1:** Relative efficiency of single- and mixed-strain designs for each type of effect. The numbers of cages are the median required amounts to achieve 80 % power in the mixed- and single-strain designs. Test statistics are based on a Mann Whitney test (*n* = 52). See Additional file 1: Tables S2–S4 for details

Effect	Median number of cages in the single-strain design (inter-quartile range)	Median number of cages needed for equivalent power in the mixed-strain design (inter-quartile range)	Test Statistics
Enrichment	252 (96 - >600)	66 (30–456)	Z = 1.74; *p* = 0.081
Strain	30 (12–108)	12 (12–42)	Z = 2.18; *p* = 0.029
Strain*Enrichment	282 (96 - >600)	120 (54–258)	Z = 1.96; *p* = 0.049

### Estimated power to detect significant Cage Type effects

The aim of this analysis was to determine if we had sufficient power to detect any effects of Cage Type that might have existed. Because *post* *hoc* power tests are inherently circular [49], we wanted to instead estimate the effect size we could have detected with 80 % power. To be conservative, and to stay consistent with our correction for multiple testing, we set the threshold for significance at p = 0.0003 for these simulations. To begin, we calculated the empirical standardized effect sizes (Cohen’s *d*, one for each dependent variable) observed in our experiment using least squared means and the standard deviations associated with these means (i.e. corrected for other factors in the model). Next, we used a binary search algorithm to calculate the effect size required to give us 80 % power to detect Cage Type effects at an alpha of 0.0003. For each outcome variable, we selected the first effect size tested by the binary search for which estimated power (based on 25,000 simulations) was not significantly different from 80 % in a binomial test using a conservative threshold of alpha = 0.2 (in practice we obtained power estimates between 79.73-80.31 %).

## Results

### Characterizing strain phenotypes

Table 2 shows all significant and trend effects of mixed- strain housing on strain differences, enrichment effects, and their interactions. Note that multiple comparison corrections, designed to reduce the Type I errors introduced by multiple testing (182 p-values were generated), reduced the significance threshold for the family of analyses to *p* = 0.0003 (see Additional file 1: Table S1 for all results for all 26 dependent variables).

**Table 2:** Significant effects (after correction for multiple comparison) for all 26 dependent variables in C57BL/6 (C), BALB/c (B), and DBA/2(D) females. All trend effects of Cage Type, i.e. whether housed in single- or mixed-strain trios, are shown in italics even though not significant after the correction. For interactions, effect directions are based on Tukey’s tests for each dependent*independent variable. Denominator degrees of freedom vary as a result of the REML procedure and some sample loss

Dependent Variable	Independent Variable	F-value	*p*-value	Direction of Effect
Normal Activity	Enrichment	F_1,110_ = 27.5	0.0001	Higher if enriched
Stereotypic Behaviour	Enrichment	F_1,115_ = 127.8	0.0001	Lower if enriched
Growth	Enrichment	F_1,143_ = 14.8	0.0001	Higher if enriched
Mean Corpuscular Volume	Enrichment	F_1,137_ = 20.7	0.0001	EH higher
Normal Activity	Strain	F_2,110_ = 10.37	0.0001	C = B > D
Stereotypic Behaviour	Strain	F_2,115_ = 9.04	0.0002	D = B > C
Novel Object Latency	Strain	F_2,161_ = 84.0	0.0001	C > B > D
Startle Response	Strain	F_2,177_ = 18.1	0.0001	B > C > D
Forced Swim Test – Duration of Floating	Strain	F_2,159_ = 144.6	0.0001	B > C > D
Forced Swim Test – Latency to Begin Floating	Strain	F_2,164_ = 86.0	0.0001	D > C = B
Faecal Corticosterone Metabolites	Strain	F_2,99_ = 57.6	0.0001	B > D > C
Spleen Weight	Strain	F_2,156_ = 35.3	0.0001	B > C = D
Growth	Strain	F_2,143_ = 21.5	0.0003	C > D > B
Mean Corpuscular Haemoglobin/Erythrocyte	Strain	F_2,146_ = 41.4	0.0001	B > C > D
Mean Corpuscular Haemoglobin Concentration	Strain	F_2,150_ = 20.7	0.0001	B > D = C
Mean Corpuscular Volume	Strain	F_2,137_ = 109.6	0.0001	C > B > D
Mean Platelet Volume	Strain	F_2,116_ = 20.4	0.0001	B = D > C
Absolute Neutrophil Count	Strain	F_2,163_ = 11.8	0.0001	C > B = D
Platelet Count	Strain	F_2,123_ = 11.1	0.0001	C > D = B
Red Blood Cell Distribution Width	Strain	F_2,120_ = 237.1	0.0001	D > B > C
Inactivity	Strain*Enrichment	F_2,105_ = 13.6	0.0001	Only D more inactive in EE
*Growth*	*Cage Type*	*F*_*1,175*_ *= 10.8*	*0.0012*	*Mixed higher*
*Forced Swim Test – Duration of Floating*	*Cage Type*	*F*_*1,159*_ *= 4.36*	*0.039*	*Mixed higher*
*Forced Swim Test – Latency to Begin Floating*	Cage Type*Enrichment	*F*_*1,164*_ *= 4.91*	*0.028*	*Post hoc tests found no significant differences between any combination*
*Absolute Neutrophil Count*	Cage Type*Enrichment	*F*_*1,163*_ *= 4.02*	*0.047*	*As above*
*Faecal Corticosterone Metabolites*	Cage Type*Strain	*F*_*2,99*_ *= 3.17*	*0.047*	*Post hoc tests found no difference within strains. Between strain differences are reduced in B and D in mixed-strain cages*

Enrichment had only a few significant main effects, while several strain differences were evident, all in expected directions. Importantly, no significant results involved Cage Type (single- or mixed-strain): there were thus no significant Cage Type effects, Cage Type*Strain interactions, or Cage Type*Enrichment interactions. The only possible Cage Type effects were trend main effects for two of the 26 variables (see Table 2); one trend for an interaction with strain for a third; and two weak trend interactions between Cage Type and the presence or absence of enrichment for two other variables. Overall, however, mixed-strain housing generally did not markedly or consistently alter animals’ phenotypes or affect the magnitudes of strain differences; and enrichment effects on all variables were also similar, regardless of whether or not mice were housed in mixed-strain trios.

### Behavioural compatibility and welfare

That mixed-strain housing did not compromise welfare was suggested by the lack of significant Cage Type effects on variables related to stress and well-being: stereotypic behaviour, novel object exploration, startle responses, latencies to begin floating, or faecal corticosterone metabolites (see Table 2). There were just two non-significant trends, and in opposing directions with respect to their potential welfare implications: mixed-strain mice tended to show longer forced swim test durations of floating, but conversely, mixed-strain mice tended to grow faster. In addition, as reported above, enrichment had similarly beneficial effects in all mice, regardless of whether housed in mixed- or single-strain trios. Furthermore, over the four months of the experiment, only three cases of barbering were observed, all taking place in single-strain, C57BL/6 cages (two in non-enriched cages, one in enriched); neither technicians nor researchers witnessed any occurrences of severe aggression; and when examinations were performed after death, no evidence of wounds was found on any mouse.

### Power to detect possible Cage Type effects

The fact that we did not find significant effects of Cage Type for any of our dependent variables raises the question: did we have the power to detect any differences that might have existed? Simple *post* *hoc* power analyses are inherently circular [49], so as an alternative way of addressing the issue we ran simulations (for each dependent variable) based on our empirical data that estimated how much larger the differences in means between single- and mixed-strain housing would have to have been for us to detect them at 80 % power and using p = 0.0003 as the threshold for significance. Our empirical effect sizes were ‘small’ [50] for all 26 dependent variables (see Table 3). The estimated required effect sizes necessary for us to have been able to likely detect an effect are all ‘large’ (see Table 3).

**Table 3:** Estimated Cage Type standardized effect sizes (Cohen’s *d*) that would be required to detect a significant effect with 80 % power (β) and a significance threshold of *p* = 0.0003 (α). Effect size calculations are based on the least squared means from the original models (i.e. they are based on transformed values that have been corrected for other factors in the model)

Dependent Variable	Empirical Effect Size (d)	Estimated Required Effect Size (d)
Normal Activity	0.09	1.24
Inactivity	0.12	1.24
Stereotypic Behaviour	0.27	1.22
Novel Object Latency	0.14	1.04
Startle Response	0.04	0.88
Forced Swim Test – Latency to Begin Floating	0.08	1.10
Forced Swim Test- Duration of Floating	0.32	1.12
Faecal Corticosterone Metabolites	0.20	0.97
Blood Glucose	0.14	0.86
Growth	0.54	2.41
Spleen Weight	0.05	1.12
White Blood Cell Count	0.29	1.21
Red Blood Cell Count	0.23	1.17
Haemoglobin	0.25	1.27
Haematocrit	0.19	1.19
Mean Corpuscular Volume	0.04	1.00
Mean Corpuscular Haemoglobin	0.05	0.93
Mean Corpuscular Haemoglobin Concentration	0.02	0.82
Red Blood Cell Distribution Width	0.10	1.18
Platelet Count	0.11	1.20
Mean Platelet Volume	0.03	1.38
Absolute Neutrophil Count	0.15	0.99
Absolute Lymphocyte Count	0.26	1.17
Absolute Monocyte Count	0.01	1.56
Absolute Eosinophil Count	0.08	1.15
Absolute Basophil Count	0.03	1.38

### Data variation within mixed- vs. Single-strain cages

The standard deviations (SD) of the measured variables, were used to measure variation within our experiment. We performed two different analyses to investigate the effects of mixing strains on the SDs of measured variables. The first was a simple sign test (one comparison for each Strain(3)*Enrichment(2)*Variable(26) combination; 156 total), which revealed significantly more cases where the SD was lower in the mixed-strain design than the single-strain design (100/156; *p* = 0.0006). As an aside, enrichment did not affect SD (80/156; *p* = 0.81) in this sign test. The second analysis was a mixed model to investigate whether mixed-strain housing predicted differences in SD, and whether any differences were due to some or all of the dependent variables. Mixed-strain housing again predicted significantly lower SDs (F_1,224_ = 17.1, *p* < 0.0001). This was consistent across all 26 dependent variables: there was no Cage Type*Variable interaction (F_25,224_ = 6.1, *p* = 0.093). Once again, a subsidiary finding was no effect of enrichment on SD (F_1,224_ = 01.5, *p* = 0.22), yet there was an Enrichment*Variable interaction (F_25,224_ = 2.42, *p* = 0.0003). A Tukey’s *post* *hoc* analysis shows that this result reflects enrichment reducing variation in stereotypic behaviour.

### Power and relative efficiency simulations

We conducted two sets of tests to compare the statistical power to detect the Strain, Enrichment and Strain*Enrichment effects offered by single- vs. mixed-strain experimental designs. The first was a paired *t*-test to compare *post hoc* power, calculated for the actual sample sizes used and the standardized effect sizes (the difference between group means divided by the pooled standard deviation [51]) observed in each experiment. This thus tested whether the various levels of observed power differed between our relatively small mixed-strain sample (17 cages by the end of the study) and the larger same-strain sample (55 cages by the end of the study). Even though the split-plot design used under a third the number of cages of the full factorial design, the levels of power achieved were not significantly different for detecting Enrichment (t_50_ = 0.15, *p* = 0.88; see Additional file 1: Figure S5), Strain (t_50_ = −0.10, *p* = 0.92; see Additional file 1: Figure S6), or Strain*Enrichment effects (t_50_ = 0.89, *p* = 0.38; Additional file 1: Figure S7).

Our second approach was to use Monte Carlo simulations to estimate relative efficiency, identifying the sample sizes required to obtain 80 % power – widely recommended as a desirably high level of power – in each type of experiment. Recognizing that experimental design may have an impact on true effect sizes, we used separate (observed) effect sizes for each experimental design (single-strain/full factorial vs. mixed-strain/split- plot) when estimating power at different sample sizes (Additional file 1: Tables S2–S4). This revealed that fewer cages were required to obtain 80 % power in the mixed-strain, split- plot design (see Table 1).

## Discussion

To validate mixed-strain housing for female laboratory mice as a potential way to reduce animal numbers without sacrificing research quality, first, we tested the hypothesis that it alters mouse phenotypes, recognizing that many researchers may be concerned that changing practice could alter well-established strain typical phenotypes and – worse – affect the magnitude or even direction of differences between strains. As in our previous study co-housing DBA/2 and C57BL/6 mice [18], results were reassuring: observed strain differences were harmonious with the literature, and their directions and magnitudes were unaffected by mixed-strain housing. Thus, DBA/2 and BALB/c mice were consistently more stereotypic than C57BL/6 mice [11, 18]; BALB/c and C57BL/6 were consistently most anxious, in behavioural tests of anxiety, followed by DBA/2 s [18, 52]; DBA/2 mice showed consistently lower durations of immobility in the forced swim test than the other two strains [52] (a variable for which there was a non-significant trend main effect of Cage Type, but not one that modified these strong strain effects); differences in growth were as expected [21] (the second variable for which there was a trend main effect of Cage Type, but again not one that modified these strong, expected strain effects); and our significant haematological results for corpuscular haemoglobin metrics, corpuscular volume, and platelet counts were largely consistent with the JAX Mouse Phenome Database, again regardless of Cage Type [53]. Faecal corticosterone metabolite outputs were also highest in BALB/c mice and lowest in C57BL/6, as previously reported [13]. This was the only one of our 26 variables for which there was even a hint of an interaction between Strain and Cage Type. However, this was again only a weak non-significant trend, and too subtle to alter the strong, highly significant, expected strain differences detected. For all other 25 variables, there was not even a trend interaction between Strain and Cage Type. Strain similarities and differences were thus well conserved in the novel housing paradigm. This means that when these strains are mixed, their phenotypes are not altered (e.g. not homogenized within cages), so that researchers can still expect to find strain typical results. This in turn also means that the value of using diverse strains is preserved, so that external validity remains high.

The second aspect of our validation investigated whether effects of a widely-used treatment, environmental enrichment, would be modified by co-housing strains. Enriched housing had several benefits expected from previous studies: enriched mice grew faster and were less stereotypic (e.g. [54]). However, for 24 of our 26 variables, mixed-strain housing did not modify, i.e. interact with, the impact of environmental enrichment. For the two others (neutrophil count and latencies to float in the forced swim test), there were weak trend interactions between Enrichment and Cage Type but these were so subtle (*post hoc* tests found no differences), and also so isolated (i.e. not accompanied by effects in biologically related variables) that we suspect they are Type I errors. Overall, mixed-strain housing thus essentially had no impacts on the effectiveness of environmental enrichment.

Our validation’s third component focused on variables related to welfare, to assess whether mixed-strain housing would compromise mouse well-being. As outlined above, for measures relating to physical health, stress, anxiety, or depression, there was no strong evidence that mixed-strain housing affected mouse welfare. The trend for mixed-strain mice to exhibit higher levels of depression-like behaviour under test is potentially worrying, but was arguably offset by the trend for improved growth, and also tenuous enough that replication is now needed to see whether it was a Type I error. Furthermore, there was no serious aggression or wounding, and minimal levels of barbering, suggesting good behavioural compatibility between all cagemates, regardless of whether housed in mixed- or single-strain trios. Facility technicians also reported that thanks to cagemates’ different colouring, cage-side inspections for mixed-strain cages were easier, faster, and more reliable (even under red light). Consequently, overall, being in a mixed-strain trio did not compromise welfare.

The fourth part of our validation was assessing the effect sizes generated by mixed-strain housing in order to ensure that our study did not fail to find obvious effects due to low power. Our empirical effect sizes were small for all 26 dependent variables suggesting that mixed-strain housing has only a small effect on phenotype overall. Therefore, the non-significant Cage Type results of the current experiment are not likely to be Type II errors; if there was a large effect of mixed-strain housing we probably could have detected it. We come to this conclusion while recognizing that what is considered a ‘meaningful difference’ is subjective and depends on various factors such as the biological significance of the measure or the accepted norms within a discipline [50].

The final aspect of our validation involved assessing variation within measured variables, to determine if mixed-strain housing increased it. Any increased variation could reduce or negate the intrinsic statistical advantages of our design, and even indicate that more animals would be needed to detect significant effects: at odds with our aim of promoting reduction. Surprisingly, we found the opposite: mixed-strain housing lowered variation, an effect seemingly consistent across our diverse variables. Our first explanation was that in mixed-strain cages, each strain perhaps occupies a set social rank, making mice within the same strain more uniform than in the single-strain design. However, the lack of Cage Type effects on strain-typical phenotypes or variables relevant to welfare makes this unlikely, and so for now, the mechanism is unknown. Regardless, this effect meant that, in addition to the inherent power benefits of this experimental design, mixed-strain housing further increased power by reducing variance in diverse types of data [55]. As a supplementary finding, just as others have recently found [56], enrichment here had no effect on variation in the data.

We then quantified the degree of power gained through this combination of experimental design and reduced variation. Our first analyses showed that the power of our final 17 mixed-strain cages for assessing effects of Enrichment, Strain, and Strain*Enrichment interactions was not significantly different from the power for 55 single-strain cages. This broadly indicates that the same results can be obtained from many fewer cages, and so many fewer mice (in this case less than a third) when a mixed-strain, split- plot design is used. Our second analysis took this a step further, using simulations based on our own data to determine the median numbers of mixed- vs. single-strain cages needed to achieve comparable levels of high power (80 %) for each dependent-independent variable combination. The number of mixed-strain cages needed was significantly lower for Strain and Strain*Enrichment effects, and tended to be lower for Enrichment effects. On average (using medians), to achieve this specified power, the split- plot design reduced the number of animals required by three quarters in analyses of Enrichment effects, and by more than half for detecting Strain and Strain*Enrichment effects. Note that both of these approaches reflect and rely on the effect sizes yielded in our particular study, and so their results should not be taken as precise guides for those planning new experiments (furthermore, for some variables, effect sizes were tiny and arguably biologically irrelevant, inflating the numbers required in those instances). However, they do demonstrate the potential of mixed- strain housing to dramatically reduce the numbers of animals used.

Such differences in required sample sizes have great ethical and economic implications. Furthermore, the intrinsic statistical benefits of this type of split-plot design are not unique to mixed-strain housing: they may apply to any case where animals of different characteristics (genotypes, phenotypes or treatment groups) live in a group to which experimental manipulations can be made (e.g. within a single cage or litter). Thus, whenever possible, this approach should be considered (after careful validation) due to its potential to reduce animal use. Around 30 million mice are used in experiments each year [57]. If using more efficient designs in this way reduced this value by just 10 % (a very conservative estimate), the numbers of mice used each year would fall by 3 million with no loss of research quality, and the cost of research would also fall. Increasing the inherent power of designs has other benefits too: it can increase the ability of small-scale studies (e.g. pilots) to generate significant results (the power benefits being most marked when sample sizes are small: see Fig. 1), and it can increase the chances of all studies generating more reliable, replicable results [58].

We do, however, acknowledge some instances in which this approach would not be beneficial or appropriate. For example, the males of our strains, or females from other strains, may not mix as well as our subjects did (e.g. [59]). Males can be difficult to socially-house because they tend to be aggressive towards their cagemates. It is currently unknown how mixing strains would affect inter-male aggression and would likely depend upon the strains in question as they differ in baseline levels of aggression [11]. Mixing strains may also significantly influence other variables other than those we measured, such as strain-specific gut microbiota [60], which could cross-contaminate and so confound some research (e.g. gastroenterological studies). Researchers not interested in female C57BL/6, DBA/2, and BALB/c mice, or the 26 variables we screened, should therefore attempt to validate mixed-strain housing (or other types of split-plot designs) for themselves, focussing on their own phenotypes and variables of interest. Furthermore, the statistical advantages of this design rely on treating cage as the unit of replication, but in some rare instances Cage may not need to be included in the model if the variables studied are known to never be affected by social factors, the physical environment, or stress (as perhaps is the case when mice serve only as donors of certain tissues for subsequent in vitro work). The statistical advantages also rely on a change in the denominator degrees of freedom substantially affecting the threshold value of F: something only manifest when there are more than two sub-units, and having diminishing returns when sample sizes are large (see Fig. 1). Finally, co-housing mice of different coat colours would not eliminate the need for marking in any project needing unique colony level identification for each individual.

## Conclusions

Using fewer laboratory animals is a clear aim of the 3Rs [1]; it also saves researchers money. Our evidence shows that mixed-strain housing can be a case where a split-plot experimental design does just that, without compromising the quality of research. At least for female C57BL/6, DBA/2, and BALB/c mice, they can be studied and housed together, so increasing external validity of the experiment, without negative implications for welfare or data variability, and while still replicating typical strain and enrichment effects. The mixed-strain housing studied here was demonstrably much more powerful than housing mice conventionally, potentially able to reduce animal numbers by half or more. Using differentially pigmented subjects, as we did, further allowed researchers and technicians to easily identify individuals in the home cage, even under red light, so obviating the use of invasive marking techniques and meeting another of the 3Rs -- refinement. Although in some cases this practice may not be appropriate, in general mixing strains in this way, or co-housing individuals of other characteristics, has great potential for reducing animal numbers by allowing the use of intrinsically powerful split- plot experimental designs.

### Ethical review

The work was approved by the University of Guelph Animal Care Committee, under the auspices of the CCAC. The Animal Use Protocol Number was 12R021/1398.

### Availability of supporting data

The data sets supporting the results of this article are available in the University of Guelph Research Data Repository: http://hdl.handle.net/10864/10939.

## Additional file

Additional file 1:
**Comprehensive list of results and addtitional information detailing the statistical benefits of a split-plot design.** (DOCX 11238 kb)

## Abbreviations

SD: Standard deviation
SH: Standard housing
EH: Enriched housing
