# Supplementary material for: Mixed-strain housing for female C57BL/6, DBA/2, and BALB/c mice: validating a split-plot design that promotes refinement and reduction
Source: BMC Med Res Methodol. 2016 Jan 27;16:11. doi: 10.1186/s12874-016-0113-7 (PMC4729181; doi:10.1186/s12874-016-0113-7)
Supplement: Additional file 1: — Comprehensive list of results and addtitional information detailing the statistical benefits of a split-plot design. (DOCX 11238 kb) [file 12874_2016_113_MOESM1_ESM.docx]

Supplementary Table 1: Complete list of results for all 26 dependent variables. Results that were significant after the correction for multiple testing (see Methods) are in bold (p≤0.0003). Results that were no longer significant after the correction for multiple testing are described as trends and are identified in italics (0.0003<p≤0.05).

| **Dependent Variable** | **Enrichment** | **Cage Type** | **Strain** | **Enrichment *Strain** | **Cage Type*Strain** | **Cage Type *Enrichment** | **Cage Type *Enrichment *Strain** |
| --- | --- | --- | --- | --- | --- | --- | --- |
| Normal Activity | **F_1,110_=27.4; p<0.0001** | F_1,110_=0.44; p=0.51 | **F_2,110_=10.4; p<0.0001** | *F_2,110_=4.7; p=0.011* | F_2,110_=0.25;  p=0.77 | F_1,110_=0.11; p=0.74 | F_2,110_=0.81; p=0.44 |
| Inactivity | *F_1,105_=12.8;*  *p=0.0005* | F_1,105_=0.86;  p=0.36 | F_2,105_=0.54;  p=0.58 | **F_2,105_=13.6;**  **p<0.0001** | F_2,105_=0.90;  p=0.41 | F_1,105_=0.36;  p=0.55 | F_2,105_=0.77;  p=0.47 |
| Stereotypic Behaviour | **F_1,115_=127.8;**  **p<0.0001** | F_1,115_=3.7;  p=0.06 | **F_2,115_=9.0**  **p=0.0002** | F_2,115_=2.5;  p=0.09 | F_2,115_=1.2;  p=0.30 | F_1,115_=0.01;  p=0.92 | F_2,115_=0.7;  p=0.50 |
| Novel Object Latency | F_1,161_=0.67;  p=0.41 | F_1,161_=0.88;  p=0.35 | **F_2,161_=83.9;**  **p<0.0001** | F_2,161_=0.30;  p=0.74 | F_2,161_=0.84;  p=0.43 | F_1,161_=0.06;  p=0.80 | F_2,161_=0.18;  p=0.84 |
| Startle Response | F_1,176_=0.03;  p=0.87 | F_1,176_=0.06;  p=0.81 | **F_2,177_=18.1;**  **p<0.0001** | F_2,179_=0.65;  p=0.52 | F_2,177_=0.21;  p=0.81 | F_1,176_=0.65;  p=0.42 | F_2,179_=0.09;  p=0.92 |
| Forced Swim Test – Latency to Begin Floating | F_1,164_=0.04;  p=0.84 | F_1,164_=0.30;  p=0.58 | **F_2,164_=86.0;**  **p<0.0001** | F_2,164_=1.72;  p=0.18 | F_2,164_=0.23;  p=0.79 | *F_1,164_=4.91;*  *p=0.03* | F_2,164_=0.84;  p=0.43 |
| Forced Swim Test – Total Duration of Floating | F_1,159_=0.08;  p=0.78 | *F_1,159_=4.36;*  *p=0.04* | **F_2,159_=144.6;**  **p<0.0001** | F_2,159_=0.82;  p=0.44 | F_2,159_=0.59;  p=0.55 | F_1,159_=1.07;  p=0.30 | F_2,159_=1.79;  p=0.17 |
| Faecal Corticosterone Metabolites | F_1,99_=0.24;  p=0.63 | F_1,99_=1.91;  p=0.17 | **F_2,99_=57.7;**  **p<0.0001** | F_2,99_=2.90;  p=0.06 | *F_2,99_=3.17;*  *p=0.047* | F_1,99_=1.37;  p=0.24 | F_2,99_=0.09;  p=0.92 |
| Blood Glucose | F_1,167_=1.70;  p=0.19 | F_1,165_=0.71;  p=0.40 | F_2,166_=1.33;  p=0.27 | *F_2,166_=5.96;*  *p=0.0032* | F_2,166_=1.92;  p=0.15 | F_1,166_=0.05;  p=0.82 | F_2,166_=0.12;  p=0.89 |
| Growth | **F_1,175_=33.9;**  **p<0.0001** | *F_1,175_=10.8;*  *p=0.0012* | **F_2,173_=8.40;**  **p=0.0003** | F_2,175_=2.19;  p=0.12 | F_2,175_=0.75;  p=0.47 | F_1,175_=3.15;  p=0.08 | F_2,175_=1.48;  p=0.23 |
| Spleen Weight | F_1,165_=0.50;  p=0.48 | F_1,159_=0.09;  p=0.77 | **F_2,156_=35.3;**  **p<0.0001** | F_2,156_=0.002;  p=0.99 | F_2,155_=1.03;  p=0.36 | F_1,156_=0.39;  p=0.53 | F_2,156_=2.51;  p=0.08 |
| White Blood Cell Count | F_1,145_=0.001;  p=0.98 | F_1,145_=3.86;  p=0.051 | F_2,145_=1.02;  p=0.36 | F_2,145_=0.49;  p=0.61 | F_2,145_=2.08;  p=0.13 | F_1,145_=1.14;  p=0.29 | F_2,145_=0.21;  p=0.81 |
| Red Blood Cell Count | *F_1,120_=5.42;*  *p=0.023* | F_1,120_=2.0;  p=0.16 | F_2,120_=1.66;  p=0.20 | F_2,120_=0.83;  p=0.44 | F_2,120_=1.11;  p=0.33 | F_1,120_=0.65;  p=0.42 | F_2,120_=0.24;  p=0.79 |
| Haemoglobin | *F_1,123_=6.89;*  *p=0.010* | F_1,123_=2.43;  p=0.12 | *F_2,123_=8.27;*  *p=0.0004* | F_2,123_=1.27;  p=0.28 | F_2,123_=0.45;  p=0.64 | F_1,123_=1.62;  p=0.21 | F_2,123_=0.30;  p=0.74 |
| Haematocrit | *F_1,121_=9.22;*  *p=0.003* | F_1,121_=1.34;  p=0.25 | *F_2,121_=6.08;*  *p=0.003* | F_2,121_=0.93;  p=0.40 | F_2,121_=1.33;  p=0.27 | F_1,121_=0.45;  p=0.50 | F_2,121_=0.20;  p=0.82 |
| Mean Corpuscular Volume | **F_1,137_=20.7;**  **p<0.0001** | F_1,137_=0.07;  p=0.80 | **F_2,137_=109.6;**  **p<0.0001** | F_2,137_=2.29;  p=0.11 | F_2,137_=1.38;  p=0.26 | F_1,137_=0.07;  p=0.79 | F_2,137_=0.22;  p=0.81 |
| Mean Corpuscular Haemoglobin | F_1,146_=0.67;  p=0.41 | F_1,146_=0.10;  p=0.75 | **F_2,146_=41.4;**  **p<0.0001** | F_2,146_=0.42;  p=0.66 | F_2,146_=0.45;  p=0.64 | F_1,146_=1.52;  p=0.22 | F_2,146_=0.69;  p=0.50 |
| Mean Corpuscular Haemoglobin Concentration | F_1,150_=1.96;  p=0.16 | F_1,150_=0.01;  p=0.91 | **F_2,150_=20.7;**  **p<0.0001** | F_2,150_=0.25;  p=0.78 | F_2,150_=1.02;  p=0.36 | F_1,150_=1.19;  p=0.28 | F_2,150_=0.09;  p=0.92 |
| Red Blood Cell Distribution Width | F_1,120_=0.03;  p=0.85 | F_1,120_=0.34;  p=0.56 | **F_2,120_=237.1;**  **p<0.0001** | *F_2,150_=3.41;*  *p=0.036* | F_2,150_=0.39;  p=0.69 | F_1,120_=1.31;  p=0.25 | F_2,120_=0.27;  p=0.777 |
| Platelet Count | F_1,123_=1.52;  p=0.22 | F_1,123_=0.43;  p=0.51 | **F_2,123_=11.1;**  **p<0.0001** | F_2,123_=0.43;  p=0.65 | F_2,123_=1.9;  p=0.15 | F_1,123_=0.96;  p=0.33 | F_2,123_=0.08;  p=0.92 |
| Mean Platelet Volume | *F_1,116_=5.41;*  *p=0.022* | F_1,116_=0.04;  p=0.83 | **F_2,116_=20.4;**  **p<0.0001** | F_2,116_=2.06;  p=0.13 | F_2,116_=0.54;  p=0.58 | F_1,116_=0.49;  p=0.48 | F_2,116_=0.09;  p=0.92 |
| Absolute Neutrophil Count | *F_1,163_=5.11;*  *p=0.025* | F_1,163_=0.94;  p=0.33 | **F_2,163_=11.8;**  **p<0.0001** | F_2,163_=0.10;  p=0.90 | F_2,163_=0.90;  p=0.41 | *F_1,163_=4.02;*  *p=0.047* | F_2,163_=0.04;  p=0.97 |
| Absolute Lymphocyte Count | F_1,149_=0.50;  p=0.48 | F_1,149_=3.20;  p=0.08 | F_2,149_=0.86;  p=0.43 | F_2,149_=0.50;  p=0.61 | F_2,149_=1.22;  p=0.30 | F_1,149_=0.03;  p=0.87 | F_2,149_=0.34;  p=0.71 |
| Absolute Monocyte Count | F_1,139_=2.70;  p=0.10 | F_1,139_=0.005;  p=0.94 | *F_2,139_=3.1;*  *p=0.049* | F_2,139_=0.53;  p=0.59 | F_2,139_=0.02;  p=0.98 | F_1,139_=3.80;  p=0.053 | F_2,139_=1.31;  p=0.27 |
| Absolute Eosinophil Count | F_1,130_=0.50;  p=0.48 | F_1,130_=0.29;  p=0.59 | F_2,130_=0.72;  p=0.49 | F_2,130_=0.63;  p=0.54 | F_2,130_=1.59;  p=0.21 | F_1,130_=2.92;  p=0.09 | F_2,130_=1.33;  p=0.27 |
| Absolute Basophil Count | F_1,122_=0.005;  p=0.94 | F_1,122_=0.02;  p=0.90 | F_2,122_=0.76;  p=0.47 | F_2,122_=1.16;  p=0.32 | F_2,122_=0.04;  p=0.96 | F_1,122_=0.12;  p=0.73 | F_2,122_=0.27;  p=0.77 |

Supplementary Table 2: Number of cages required to achieve 80% power for detecting Enrichment effects across all 26 dependent variables. Effect sizes have been calculated separately for single- and mixed-strain designs, based on our own results.

|  | **Single-strain Design** | | **Mixed-strain Design** | |
| --- | --- | --- | --- | --- |
| **Dependent Variable** | **Cages** | **Effect Size (η_p_^2^)** | **Cages** | **Effect Size (η_p_^2^)** |
| Normal Activity | 30 | 0.288 | 24 | 0.338 |
| Inactivity | 48 | 0.16 | 66 | 0.126 |
| Stereotypic Behaviour | 12 | 0.677 | 12 | 0.646 |
| Novel Object Latency | 426 | 0.032 | > 600 | 0.009 |
| Startle Response | 444 | 0.022 | > 600 | 0.007 |
| Forced Swim Test – Latency to Begin Floating | 96 | 0.077 | 66 | 0.126 |
| Forced Swim Test- Duration of Floating | 324 | 0.022 | 456 | 0.019 |
| Faecal Corticosterone Metabolites | 156 | 0.02 | > 600 | 0.007 |
| Blood Glucose | 186 | 0.044 | 402 | 0.022 |
| Growth | 108 | 0.095 | 18 | 0.385 |
| Spleen Weight | > 600 | 0.003 | 84 | 0.099 |
| White Blood Cell Count | 534 | 0.016 | 216 | 0.042 |
| Red Blood Cell Count | 198 | 0.034 | 42 | 0.188 |
| Haemoglobin | 300 | 0.03 | 24 | 0.31 |
| Haematocrit | 84 | 0.088 | 30 | 0.255 |
| Mean Corpuscular Volume | 30 | 0.294 | 24 | 0.375 |
| Mean Corpuscular Haemoglobin | > 600 | 0.0001 | 42 | 0.187 |
| Mean Corpuscular Haemoglobin Concentration | 72 | 0.08 | > 600 | 0.009 |
| Red Blood Cell Distribution Width | 408 | 0.023 | 270 | 0.006 |
| Platelet Count | > 600 | 0.001 | 60 | 0.149 |
| Mean Platelet Volume | 204 | 0.034 | 36 | 0.232 |
| Absolute Neutrophil Count | > 600 | 0.004 | 24 | 0.337 |
| Absolute Lymphocyte Count | > 600 | 0.013 | > 600 | 0.011 |
| Absolute Monocyte Count | > 600 | 0.0009 | 30 | 0.285 |
| Absolute Eosinophil Count | 102 | 0.091 | 276 | 0.031 |
| Absolute Basophil Count | > 600 | 0.001 | > 600 | 0.005 |

Supplementary Table 3: Number of cages required to achieve 80% power for detecting Strain effects across all 26 dependent variables. Effect sizes have been calculated separately for single- and mixed-strain designs, based on our own results.

.

|  | **Single-strain Design** | | **Mixed-strain Design** | |
| --- | --- | --- | --- | --- |
| **Dependent Variable** | **Cages** | **Effect Size (η_p_^2^)** | **Cages** | **Effect Size (η_p_^2^)** |
| Normal Activity | 42 | 0.243 | 18 | 0.238 |
| Inactivity | 570 | 0.016 | 48 | 0.091 |
| Stereotypic Behaviour | 30 | 0.320 | 42 | 0.138 |
| Novel Object Latency | 12 | 0.731 | 12 | 0.723 |
| Startle Response | 24 | 0.392 | 12 | 0.368 |
| Forced Swim Test – Latency to Begin Floating | 12 | 0.723 | 12 | 0.795 |
| Forced Swim Test- Duration of Floating | 12 | 0.805 | 12 | 0.866 |
| Faecal Corticosterone Metabolites | 12 | 0.745 | 12 | 0.474 |
| Blood Glucose | 522 | 0.021 | 42 | 0.117 |
| Growth | 24 | 0.367 | 12 | 0.458 |
| Spleen Weight | 24 | 0.406 | 12 | 0.497 |
| White Blood Cell Count | 96 | 0.104 | 72 | 0.075 |
| Red Blood Cell Count | 84 | 0.109 | 102 | 0.053 |
| Haemoglobin | 66 | 0.142 | 12 | 0.364 |
| Haematocrit | 144 | 0.065 | 18 | 0.270 |
| Mean Corpuscular Volume | 12 | 0.772 | 12 | 0.793 |
| Mean Corpuscular Haemoglobin | 12 | 0.607 | 12 | 0.680 |
| Mean Corpuscular Haemoglobin Concentration | 18 | 0.417 | 12 | 0.479 |
| Red Blood Cell Distribution Width | 12 | 0.902 | 12 | 0.873 |
| Platelet Count | 84 | 0.109 | 12 | 0.418 |
| Mean Platelet Volume | 30 | 0.300 | 12 | 0.461 |
| Absolute Neutrophil Count | 24 | 0.400 | 30 | 0.169 |
| Absolute Lymphocyte Count | > 600 | 0.007 | 42 | 0.132 |
| Absolute Monocyte Count | 108 | 0.093 | 66 | 0.077 |
| Absolute Eosinophil Count | > 600 | 0.008 | 42 | 0.132 |
| Absolute Basophil Count | 282 | 0.041 | 312 | 0.016 |

Supplementary Table 4: Number of cages required to achieve 80% power for detecting Strain*Enrichment effects across all 26 dependent variables. Effect sizes have been calculated separately for single- and mixed-strain designs, based on our own results.

|  | **Single-strain Design** | | **Mixed-strain Design** | |
| --- | --- | --- | --- | --- |
| **Dependent Variable** | **Cages** | **Effect Size (η_p_^2^)** | **Cages** | **Effect Size (η_p_^2^)** |
| Normal Activity | 96 | 0.110 | 24 | 0.215 |
| Inactivity | 30 | 0.323 | 18 | 0.338 |
| Stereotypic Behaviour | 72 | 0.140 | 168 | 0.033 |
| Novel Object Latency | > 600 | 0.007 | 258 | 0.021 |
| Startle Response | 222 | 0.051 | > 600 | 0.008 |
| Forced Swim Test – Latency to Begin Floating | > 600 | 0.007 | 30 | 0.171 |
| Forced Swim Test- Duration of Floating | > 600 | 0.006 | 30 | 0.163 |
| Faecal Corticosterone Metabolites | 72 | 0.139 | 150 | 0.034 |
| Blood Glucose | 60 | 0.165 | 36 | 0.152 |
| Growth | 420 | 0.026 | 120 | 0.046 |
| Spleen Weight | 150 | 0.068 | > 600 | 0.002 |
| White Blood Cell Count | > 600 | 0.011 | 138 | 0.039 |
| Red Blood Cell Count | > 600 | 0.007 | 102 | 0.052 |
| Haemoglobin | 336 | 0.030 | 54 | 0.094 |
| Haematocrit | > 600 | 0.016 | 102 | 0.049 |
| Mean Corpuscular Volume | 78 | 0.126 | 120 | 0.044 |
| Mean Corpuscular Haemoglobin | 132 | 0.065 | > 600 | 0.003 |
| Mean Corpuscular Haemoglobin Concentration | 420 | 0.018 | > 600 | 0.002 |
| Red Blood Cell Distribution Width | 72 | 0.110 | 54 | 0.093 |
| Platelet Count | > 600 | 0.005 | 186 | 0.028 |
| Mean Platelet Volume | 138 | 0.068 | 96 | 0.055 |
| Absolute Neutrophil Count | > 600 | 0.009 | > 600 | 0.0007 |
| Absolute Lymphocyte Count | > 600 | 0.018 | 132 | 0.040 |
| Absolute Monocyte Count | > 600 | 0.006 | 54 | 0.088 |
| Absolute Eosinophil Count | 102 | 0.103 | 390 | 0.015 |
| Absolute Basophil Count | 228 | 0.046 | 96 | 0.054 |


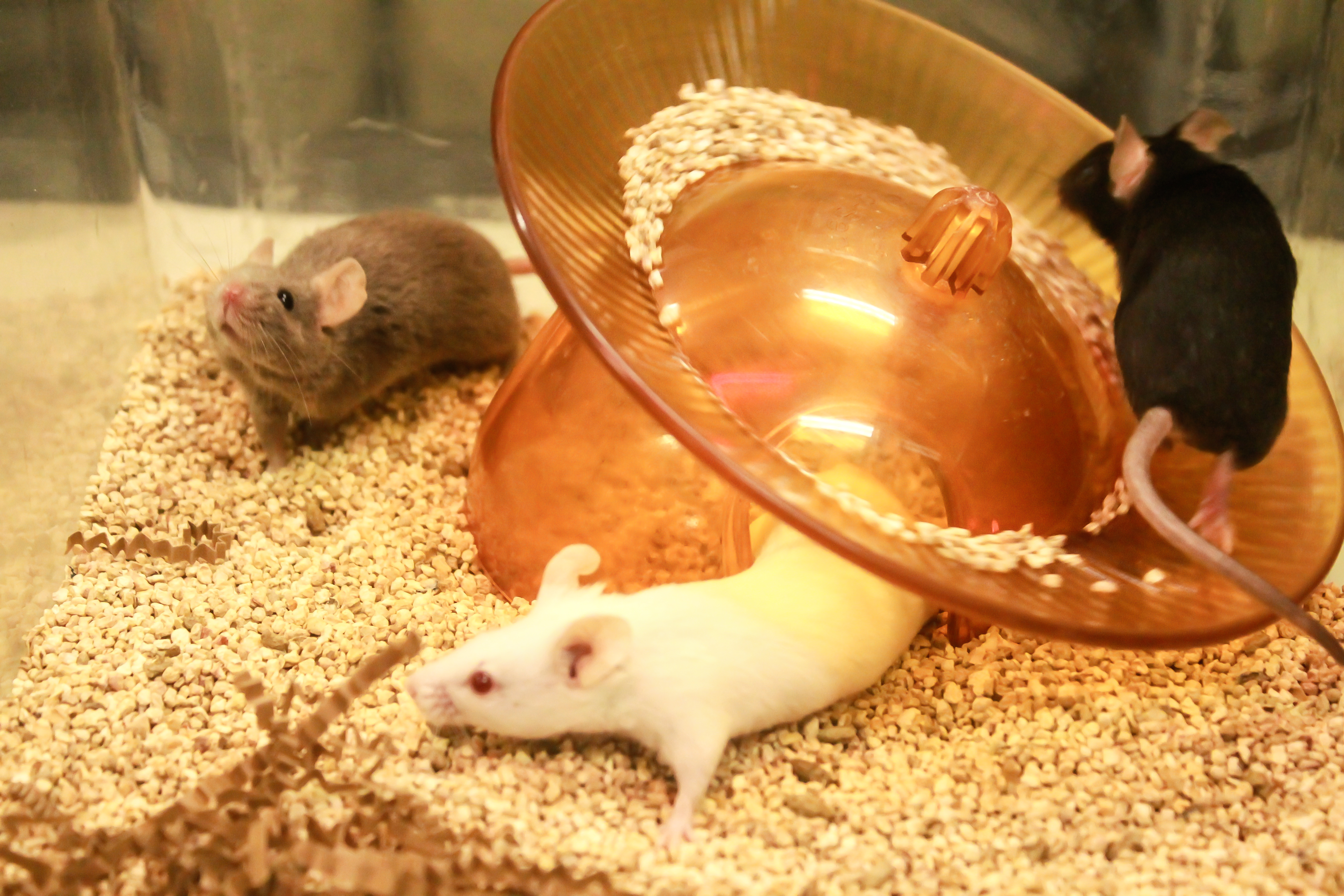


Supplementary Figure 1: The contrast in coat colours between the three strains: C57BL/6 (black), BALB/c (white), and DBA/2 (brown); also shown is one of the enrichment items used in the enriched condition: an igloo and ‘fast-trac’ wheel combo by Bio Serv®.

Single Strain Groups

Mixed Strain Groups

n = 36

Supplementary Figure 2: Hypothetical experimental designs comparing single- and mixed-strain housing for 36 mice in each design. Coloured circles represent different strains and the light- and dark-blue backgrounds represent different treatments.

**Derivation of the denominator degrees of freedom**

As long as three or more strains are used, the resulting split-plot nature of the experimental design will increase intrinsic statistical power, as follows. Suppose the aim is to test the effect of a treatment compared to a control condition, on three strains, with all mice in a single cage receiving the same treatment (e.g. environmental enrichment; presence/absence of crowding; diet type, etc.). A typical experiment might house three mice in each cage, all from the same strain. Thus there might be 12 cages in total (36 mice), with two replicate cages for each of the six strain-by-treatment combinations. In our alternative ‘mixed-strain’ design, in contrast, one mouse of each strain is housed in each cage, yielding six replicate cages for each treatment, each cage containing one mouse from each strain (see supplementary Fig. 2 for a pictorial representation).

The single-strain, full factorial design uses the same denominator mean square for all F-ratios. Specifically it would be , where is the number of cages nested in each of the treatment by strain combinations, *t* is the number of levels of the treatment, and *s* is the number of strains. The number of cages used must be divisible by the number of treatment by strain combinations, 6 in this example, to yield a balanced design. can then be simplified as .

Conversely, the mixed-strain, split-plot design uses two different denominator mean squares for the F-ratios (the ‘whole plot error’ and the ‘sub-plot error’). Firstly, for testing treatment effects (the ‘whole plot’) it would be where is the number of cages nested in each level of the treatment, and *t* is again the number of levels of the treatment. The number of cages used must be divisible by the number of treatment levels for a balanced design, 2 in this case. So can be simplified as . Secondly, for testing strain (the ‘sub-plot’) and the strain by treatment interaction, it would be which can be simplified in this case to.


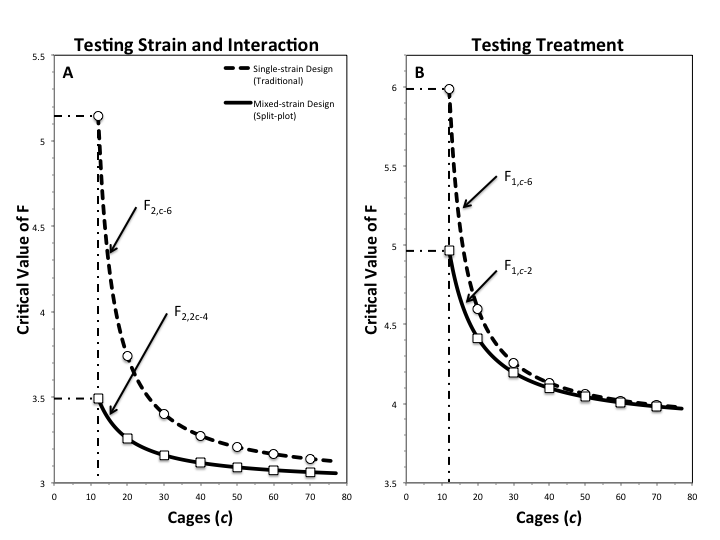


Supplementary Figure 3: Shown are the critical values of *F* needed to reject the null hypothesis at the α=0.05 level for two hypothetical experiments testing the effects of a treatment vs. a control on three strains of mice. Smaller values for the critical *F* imply greater statistical power. Highlighted here are the substantially lower critical *F*-values for mixed-strain designs using 12 cages as described in Supplementary Fig. 2. The absolute difference in the critical value of *F* between designs declines with increasing sample size and thus the power benefits are most pronounced at smaller sample sizes in both A and B.


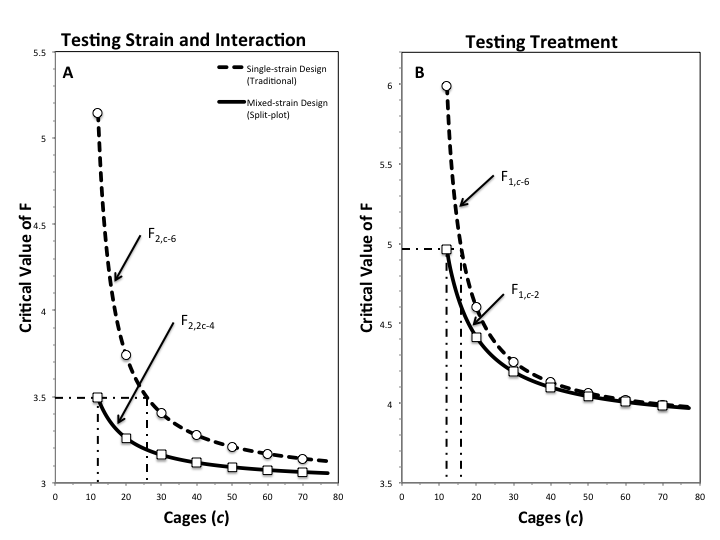


Supplementary Figure 4: Shown are the critical values of *F* needed to reject the null hypothesis at the α=0.05 level for two hypothetical experiments testing the effects of a treatment vs. a control on three strains of mice. Smaller values for the critical *F* imply greater statistical power. Highlighted here are the substantially fewer numbers of cages needed in the mixed-strain design to achieve the same critical values of *F* assuming 12 mixed-strain cages are used. In A, the relative difference in the number of cages needed to achieve the same critical value of F is constant: 2*c*+2 single-strain cages equal *c* mixed-strain cages. In B, the number of cages required to achieve the same critical value of *F* converges quickly between designs.

Supplementary Figure 5:

Mean (±SE) estimated power to detect enrichment effects on our 26 variables in our mixed-strain (white bar; *c*=17) and single-strain (shaded bar; *c*=55) groups, where c = total number of cages across both enrichment groups (each cage holding three mice). The similarity in power between the two designs, despite the substantially smaller number of cages and mice used in the mixed strain design, is clear (see also Supp. Figs. 6 and 7).

Supplementary Figure 6:

Mean (±SE) estimated power to detect strain effects on our 26 variables in our mixed-strain (white bar; *c*=17) and single-strain (shaded bar; *c*=55) groups, where c = total number of cages housing the three strains.

Supplementary Figure 7:

Mean (±SE) estimated power to detect enrichment-by-strain interaction effects on our 26 variables in our mixed-strain (white bar; *c*=17) and single-strain (shaded bar; *c*=55) groups, where c = total number of cages across all strain*housing treatments.
